# (*E*)-4-{2-[(2-Hy­droxy­naphthalen-1-yl)methyl­idene]hydrazinecarbon­yl}pyridinium nitrate

**DOI:** 10.1107/S160053681200061X

**Published:** 2012-01-11

**Authors:** Rahman Bikas, Hassan Hosseini Monfared, Tadeusz Lis, Milosz Siczek

**Affiliations:** a Young Researchers Club, Tabriz Branch, Islamic Azad University, Tabriz, Iran; b Department of Chemistry, University of Zanjan, 45195-313, Zanjan, Iran; c Faculty of Chemistry, University of Wroclaw, Joliot-Curie 14, Wroclaw 50-383, Poland

## Abstract

The title compound, C_17_H_14_N_3_O_2_^+^·NO_3_^−^, is an aroylhydrazone-based material consisting of a 4-(hydrazinecarbon­yl)pyridinium cation and a nitrate anion. In the cation, the dihedral angle between the benzene ring and the naphthalene ring system is 2.20 (7)°. In the cation, the configuration about the C=N bond is *E*. There is an intra­molecular O—H⋯N hydrogen bond in the cation, and the supra­molecular structure is stabilized by inter­molecular N—H⋯O hydrogen bonds and weak C—H⋯O contacts between the cation and the nitrate anion.

## Related literature

For historical background to aroylhydrazones, see: Craliz *et al.* (1955 ▶). For related structures see: Bikas *et al.* (2010*a* ▶,*b* ▶); Hosseini Monfared *et al.* (2010*a* ▶); Abdel-Aziz *et al.* (2011 ▶). For background to the development of hydrazide derivatives for biological evaluation, see: Carvalho *et al.* (2008 ▶). For catalytic applications of aroylhydrazones, see: Hosseini Monfared *et al.* (2010*b* ▶). The overall structure of the cation is very similar to that found for free ligand, see: Richardson & Bernhardt (1999 ▶).
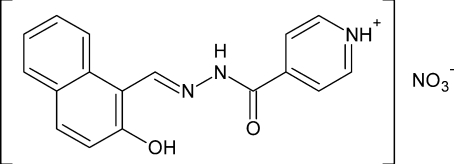


## Experimental

### 

#### Crystal data

C_17_H_14_N_3_O_2_^+^·NO_3_^−^
*M* *_r_* = 354.32
Monoclinic, 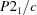

*a* = 8.695 (3) Å
*b* = 6.375 (2) Å
*c* = 28.955 (9) Å
β = 98.19 (4)°
*V* = 1588.6 (9) Å^3^
*Z* = 4
Mo *K*α radiation
μ = 0.11 mm^−1^
*T* = 100 K
0.30 × 0.10 × 0.07 mm

#### Data collection

Oxford Diffraction Xcalibur PX kappa-geometry diffractometer with an Onyx CCD camera
12608 measured reflections
5046 independent reflections
3368 reflections with *I* > 2σ(*I*)
*R*_int_ = 0.030

#### Refinement

*R*[*F* ^2^ > 2σ(*F* ^2^)] = 0.040
*wR*(*F* ^2^) = 0.102
*S* = 1.03
5046 reflections
236 parameters
H-atom parameters constrained
Δρ_max_ = 0.41 e Å^−3^
Δρ_min_ = −0.22 e Å^−3^

### 

Data collection: *CrysAlis CCD* (Oxford Diffraction, 2003 ▶); cell refinement: *CrysAlis RED* (Oxford Diffraction, 2003 ▶); data reduction: *CrysAlis RED*; program(s) used to solve structure: *SHELXS97* (Sheldrick, 2008 ▶); program(s) used to refine structure: *SHELXL97* (Sheldrick, 2008 ▶); molecular graphics: *DIAMOND* (Brandenburg, 2006 ▶); software used to prepare material for publication: *publCIF* (Westrip, 2010 ▶).

## Supplementary Material

Crystal structure: contains datablock(s) I, global. DOI: 10.1107/S160053681200061X/go2040sup1.cif

Structure factors: contains datablock(s) I. DOI: 10.1107/S160053681200061X/go2040Isup2.hkl

Supplementary material file. DOI: 10.1107/S160053681200061X/go2040Isup3.cml

Additional supplementary materials:  crystallographic information; 3D view; checkCIF report

## Figures and Tables

**Table 1:** Hydrogen-bond geometry (Å, °)

*D*—H⋯*A*	*D*—H	H⋯*A*	*D*⋯*A*	*D*—H⋯*A*
O1—H1⋯N1	0.84	1.82	2.5519 (15)	145
N2—H2⋯O3*A*	0.88	2.21	3.0332 (19)	155
N3—H3*A*⋯O1*A*^i^	0.88	1.80	2.6794 (14)	174
C14—H14⋯O3*A*	0.95	2.26	3.1528 (16)	156
C8—H8⋯O2^ii^	0.95	2.60	3.2449 (19)	125
C15—H15⋯O2*A*^iii^	0.95	2.61	3.3089 (19)	130
C16—H16⋯O1*A*^iv^	0.95	2.28	3.1923 (16)	160
C16—H16⋯O2*A*^iv^	0.95	2.62	3.4553 (19)	147

Symmetry codes: (i) 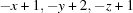; (ii) 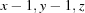; (iii) 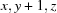; (iv) 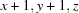.

**Fig. 1.**
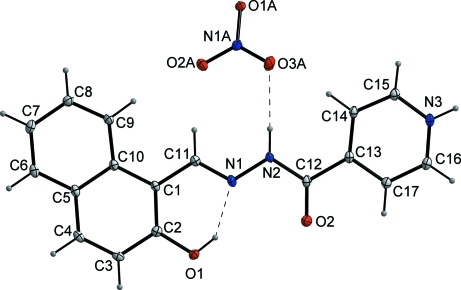
The molecular structure of the title compound with labelling scheme and anisotropic displacement ellipsoids (drawn at 30% probability level for non-H atoms).

**Fig. 2.**
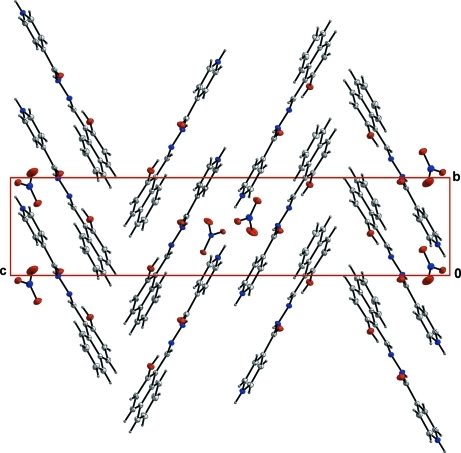
The packing diagram of the title compound.

**Fig. 3.**
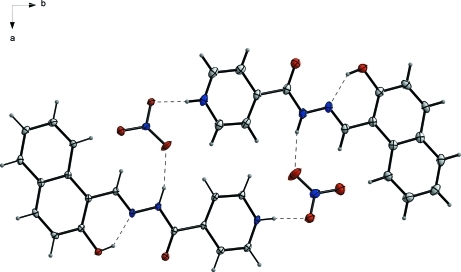
A diagram showing formation of intra- (O–H···N) and intermolecular (N–H···O) hydrogen bonds between anions and cations.

## supplementary crystallographic information

### Comment

Hydrazone ligands, a class of Schiff base, derived from the condensation of acid hydrazides (R–CO–NH–NH_2_) with aromatic 2-hydroxy carbonyl compounds are important tridentate O, N, O-donor ligands. As biologically active compounds, hydrazones find application in the treatment of diseases such as anti-tumor, tuberculosis, leprosy and mental disorder. Hydrazone ligands create environment similar to biological systems by usually making coordination through oxygen and nitrogen atoms. Furthermore hydrazones have wide spread applications in fields such as coordination chemistry, bioinorganic chemistry, in magnetic, electronic, nonlinear optically active and fluorescent compounds. Aroylhydrazone complexes seem to be a good candidate for catalytic oxidation studies because of their resist to oxidation (Hosseini Monfared *et al.*, 2010*b*).

As part of our studies on the synthesis and characterization of hydrazone derivatives, we report here the crystal structure of (*E*)-4-(2-((2-hydroxynaphthalen-1-yl)methylene)hydrazinecarbonyl)pyridinium nitrate. The asymmetric unit of C_17_H_14_N_4_O_5_, consists of a (*E*)-4-(2-((2-hydroxynaphthalen-1-yl)methylene)hydrazinecarbonyl)pyridinium cation and a nitrate anion (Fig. 1). The dihedral angle between the mean planes of the benzene and naphthalene rings is 2.20 (7)°. The cation displays a *trans* configuration with respect to the C=N and N—N bonds. It is to note that the overall structure of the cation is very similar to that found for free ligand Richardson & Bernhardt (1999). The packing diagram of the title compound is shown in Fig. 2. There is a strong intramolecular O—H···N hydrogen bond in which the N of the azomethine group (–C=N–) acts as hydrogen acceptor for the hydrogen O—H group attached to the naphthalene ring. Two intermolecular N—H···O hydrogen bonds are formed between cation and anion where NO_3_^-^ acts as hydrogen bonds acceptor (Fig. 3). The supramolecular structure is further stabilised by C—H···O interactions.

### Experimental

All reagents were commercially available and used as received. A methanol (10 ml) solution of 2-hydroxy-1-naphthaldehyde (1.63 mmol) was dropwise added to a methanol solution (10 ml) of 4-pyridine carboxylic acid hydrazide (1.63 mmol), and the mixture was refluxed for 3 hrs. Then the solution was evaporated on a steam bath to 5 ml and cooled to room temperature. The resultant yellow precipitate was separated and filtered off, washed with 5 ml of cooled methanol and then dried in air. 1 mmol of this solid was placed in one arm of a branched tube with 2 mmol of Mn(NO_3_)_2_.4H_2_O. Methanol was carefully added to fill the arms, the tube was sealed and the arm containing the reagents was immersed in an oil bath at 60 °C while the other arm was kept at ambient temperature. After 4 days, crystals were deposited in the cooler arm, which were filtered off and air dried. Yield: 85%, Selected IR spectrum: 3430 (s, broad), 1630 (*s*), 1600 (*m*), 1549 (*m*), 1384 (*versus*), 1291 (*m*), 972 (*m*), 834 (*s*), 764 cm^-1^ (*m*).

### Refinement

The hydrogen atoms of the N—H and O—H groups were positioned geometrically and refined as riding atoms with, N—H = 0.88 Å and *U*_iso_(H) = 1.2 *U*_eq_(N), O—H = 0.84 Å and *U*_iso_(H) = 1.5 *U*_eq_(O). The C—H hydrogen atoms were positioned geometrically and refined as riding atoms with C—H = 0.95 Å and *U*_iso_(H) = 1.2 *U*_eq_(C).

### Crystal data

**Table:**

C_17_H_14_N_3_O_2_^+^·NO_3_^−^	*F*(000) = 736
*M**_r_* = 354.32	*D*_x_ = 1.481 Mg m^−^^3^
Monoclinic, *P*2_1_/*c*	Mo *K*α radiation, λ = 0.71073 Å
Hall symbol: -P 2ybc	Cell parameters from 4462 reflections
*a* = 8.695 (3) Å	θ = 2–70°
*b* = 6.375 (2) Å	µ = 0.11 mm^−^^1^
*c* = 28.955 (9) Å	*T* = 100 K
β = 98.19 (4)°	Needle, orange
*V* = 1588.6 (9) Å^3^	0.30 × 0.10 × 0.07 mm
*Z* = 4	

### Data collection

**Table:**

Oxford Diffraction Xcalibur PX kappa-geometry diffractometer with an Onyx CCD camera	3368 reflections with *I* > 2σ(*I*)
Radiation source: fine-focus sealed tube	*R*_int_ = 0.030
graphite	θ_max_ = 31.1°, θ_min_ = 2.6°
ω and phi scans	*h* = −12→12
12608 measured reflections	*k* = −8→9
5046 independent reflections	*l* = −42→35

### Refinement

**Table:**

Refinement on *F*^2^	Primary atom site location: structure-invariant direct methods
Least-squares matrix: full	Secondary atom site location: difference Fourier map
*R*[*F*^2^ > 2σ(*F*^2^)] = 0.040	Hydrogen site location: inferred from neighbouring sites
*wR*(*F*^2^) = 0.102	H-atom parameters constrained
*S* = 1.03	*w* = 1/[σ^2^(*F*_o_^2^) + (0.048*P*)^2^] where *P* = (*F*_o_^2^ + 2*F*_c_^2^)/3
5046 reflections	(Δ/σ)_max_ = 0.001
236 parameters	Δρ_max_ = 0.41 e Å^−^^3^
0 restraints	Δρ_min_ = −0.22 e Å^−^^3^

### Special details

**Table:**

Geometry. All e.s.d.'s (except the e.s.d. in the dihedral angle between two l.s. planes) are estimated using the full covariance matrix. The cell e.s.d.'s are taken into account individually in the estimation of e.s.d.'s in distances, angles and torsion angles; correlations between e.s.d.'s in cell parameters are only used when they are defined by crystal symmetry. An approximate (isotropic) treatment of cell e.s.d.'s is used for estimating e.s.d.'s involving l.s. planes.
Refinement. Refinement of *F*^2^ against ALL reflections. The weighted *R*-factor *wR* and goodness of fit *S* are based on *F*^2^, conventional *R*-factors *R* are based on *F*, with *F* set to zero for negative *F*^2^. The threshold expression of *F*^2^ > σ(*F*^2^) is used only for calculating *R*-factors(gt) *etc*. and is not relevant to the choice of reflections for refinement. *R*-factors based on *F*^2^ are statistically about twice as large as those based on *F*, and *R*- factors based on ALL data will be even larger.

### Fractional atomic coordinates and isotropic or equivalent isotropic displacement parameters (Å^2^)

**Table:**

	*x*	*y*	*z*	*U*_iso_*/*U*_eq_	
O1	0.97012 (9)	0.07072 (13)	0.68081 (3)	0.02035 (19)	
H1	0.9395	0.1749	0.6642	0.031*	
C1	0.69254 (13)	0.00322 (16)	0.67027 (4)	0.0134 (2)	
C2	0.84656 (13)	−0.04806 (17)	0.68768 (4)	0.0157 (2)	
C3	0.88242 (14)	−0.23367 (17)	0.71408 (4)	0.0183 (2)	
H3	0.9873	−0.2643	0.7263	0.022*	
C4	0.76730 (14)	−0.36810 (17)	0.72201 (4)	0.0182 (2)	
H4	0.7931	−0.4913	0.7399	0.022*	
C5	0.60945 (14)	−0.32776 (17)	0.70405 (4)	0.0160 (2)	
C6	0.49134 (15)	−0.47202 (18)	0.71132 (4)	0.0196 (3)	
H6	0.5186	−0.5988	0.7277	0.023*	
C7	0.33824 (15)	−0.43169 (19)	0.69510 (5)	0.0229 (3)	
H7	0.2600	−0.5301	0.6998	0.028*	
C8	0.29866 (14)	−0.2422 (2)	0.67130 (4)	0.0229 (3)	
H8	0.1924	−0.2118	0.6608	0.028*	
C9	0.41042 (14)	−0.10038 (18)	0.66288 (4)	0.0189 (2)	
H9	0.3803	0.0250	0.6463	0.023*	
C10	0.57040 (13)	−0.13845 (16)	0.67860 (4)	0.0143 (2)	
C11	0.65724 (13)	0.19520 (17)	0.64348 (4)	0.0149 (2)	
H11	0.5526	0.2348	0.6332	0.018*	
N1	0.77034 (11)	0.31000 (14)	0.63397 (3)	0.0160 (2)	
N2	0.73812 (12)	0.49164 (14)	0.60861 (3)	0.0163 (2)	
H2	0.6425	0.5334	0.5989	0.020*	
C12	0.86518 (13)	0.60206 (17)	0.59968 (4)	0.0166 (2)	
O2	0.99749 (10)	0.53944 (13)	0.61115 (3)	0.0244 (2)	
C13	0.83618 (13)	0.80954 (16)	0.57479 (4)	0.0151 (2)	
C14	0.69062 (14)	0.88961 (17)	0.55696 (4)	0.0172 (2)	
H14	0.5987	0.8157	0.5611	0.021*	
C15	0.68192 (14)	1.07777 (17)	0.53318 (4)	0.0181 (2)	
H15	0.5834	1.1331	0.5205	0.022*	
N3	0.81220 (12)	1.18318 (14)	0.52782 (3)	0.0178 (2)	
H3A	0.8043	1.3007	0.5117	0.021*	
C16	0.95343 (14)	1.11540 (18)	0.54613 (4)	0.0198 (3)	
H16	1.0429	1.1970	0.5431	0.024*	
C17	0.96832 (14)	0.92589 (18)	0.56950 (4)	0.0188 (2)	
H17	1.0684	0.8750	0.5819	0.023*	
N1A	0.32288 (12)	0.41051 (15)	0.54333 (4)	0.0204 (2)	
O1A	0.19647 (10)	0.44786 (12)	0.51679 (3)	0.0205 (2)	
O2A	0.34501 (10)	0.23488 (14)	0.56160 (3)	0.0273 (2)	
O3A	0.42367 (12)	0.55145 (16)	0.54972 (4)	0.0453 (3)	

### Atomic displacement parameters (Å^2^)

**Table:**

	*U*^11^	*U*^22^	*U*^33^	*U*^12^	*U*^13^	*U*^23^
O1	0.0168 (4)	0.0201 (4)	0.0232 (5)	−0.0038 (3)	−0.0003 (3)	0.0040 (3)
C1	0.0172 (5)	0.0125 (5)	0.0104 (5)	0.0002 (4)	0.0017 (4)	−0.0002 (4)
C2	0.0177 (5)	0.0165 (5)	0.0127 (6)	−0.0016 (4)	0.0017 (4)	−0.0028 (4)
C3	0.0187 (5)	0.0203 (5)	0.0150 (6)	0.0039 (5)	−0.0004 (4)	0.0008 (5)
C4	0.0249 (6)	0.0159 (5)	0.0137 (6)	0.0046 (5)	0.0025 (5)	0.0028 (4)
C5	0.0225 (6)	0.0144 (5)	0.0117 (6)	0.0010 (4)	0.0050 (4)	0.0001 (4)
C6	0.0274 (6)	0.0158 (5)	0.0166 (6)	−0.0003 (5)	0.0067 (5)	0.0021 (5)
C7	0.0239 (6)	0.0231 (6)	0.0232 (7)	−0.0070 (5)	0.0081 (5)	0.0024 (5)
C8	0.0177 (6)	0.0284 (6)	0.0221 (7)	−0.0032 (5)	0.0009 (5)	0.0031 (5)
C9	0.0192 (5)	0.0191 (5)	0.0177 (6)	−0.0001 (5)	0.0006 (5)	0.0051 (5)
C10	0.0175 (5)	0.0144 (5)	0.0113 (6)	−0.0002 (4)	0.0027 (4)	−0.0003 (4)
C11	0.0180 (5)	0.0135 (5)	0.0128 (6)	0.0000 (4)	0.0014 (4)	−0.0008 (4)
N1	0.0217 (5)	0.0116 (4)	0.0144 (5)	−0.0010 (4)	0.0020 (4)	0.0012 (4)
N2	0.0192 (5)	0.0128 (4)	0.0168 (5)	−0.0009 (4)	0.0018 (4)	0.0038 (4)
C12	0.0210 (6)	0.0139 (5)	0.0147 (6)	−0.0037 (4)	0.0021 (4)	−0.0015 (4)
O2	0.0194 (4)	0.0202 (4)	0.0327 (6)	−0.0015 (3)	0.0008 (4)	0.0044 (4)
C13	0.0201 (5)	0.0132 (5)	0.0122 (6)	−0.0030 (4)	0.0033 (4)	−0.0009 (4)
C14	0.0200 (5)	0.0153 (5)	0.0160 (6)	−0.0057 (4)	0.0019 (4)	−0.0007 (4)
C15	0.0210 (6)	0.0167 (5)	0.0162 (6)	−0.0030 (5)	0.0014 (5)	−0.0010 (4)
N3	0.0249 (5)	0.0146 (4)	0.0141 (5)	−0.0048 (4)	0.0030 (4)	0.0016 (4)
C16	0.0212 (6)	0.0194 (5)	0.0193 (6)	−0.0064 (5)	0.0051 (5)	−0.0002 (5)
C17	0.0193 (6)	0.0192 (5)	0.0178 (6)	−0.0033 (5)	0.0025 (5)	0.0006 (5)
N1A	0.0182 (5)	0.0218 (5)	0.0207 (6)	−0.0045 (4)	0.0011 (4)	0.0028 (4)
O1A	0.0169 (4)	0.0208 (4)	0.0223 (5)	−0.0037 (3)	−0.0020 (3)	0.0052 (3)
O2A	0.0274 (5)	0.0225 (4)	0.0304 (6)	0.0001 (4)	−0.0009 (4)	0.0091 (4)
O3A	0.0312 (6)	0.0385 (6)	0.0590 (8)	−0.0226 (5)	−0.0178 (5)	0.0197 (5)

### Geometric parameters (Å, °)

**Table:**

O1—C2	1.3521 (14)	C11—H11	0.9500
O1—H1	0.8400	N1—N2	1.3784 (13)
C1—C2	1.4007 (16)	N2—C12	1.3654 (15)
C1—C10	1.4404 (16)	N2—H2	0.8800
C1—C11	1.4581 (16)	C12—O2	1.2183 (14)
C2—C3	1.4192 (16)	C12—C13	1.5101 (16)
C3—C4	1.3618 (17)	C13—C14	1.3938 (17)
C3—H3	0.9500	C13—C17	1.3942 (16)
C4—C5	1.4202 (17)	C14—C15	1.3799 (16)
C4—H4	0.9500	C14—H14	0.9500
C5—C6	1.4165 (17)	C15—N3	1.3449 (15)
C5—C10	1.4295 (16)	C15—H15	0.9500
C6—C7	1.3714 (18)	N3—C16	1.3380 (16)
C6—H6	0.9500	N3—H3A	0.8800
C7—C8	1.4091 (18)	C16—C17	1.3818 (17)
C7—H7	0.9500	C16—H16	0.9500
C8—C9	1.3741 (17)	C17—H17	0.9500
C8—H8	0.9500	N1A—O2A	1.2416 (13)
C9—C10	1.4215 (16)	N1A—O3A	1.2503 (13)
C9—H9	0.9500	N1A—O1A	1.2706 (13)
C11—N1	1.2867 (15)		
			
C2—O1—H1	109.5	N1—C11—C1	118.81 (10)
C2—C1—C10	118.90 (10)	N1—C11—H11	120.6
C2—C1—C11	120.36 (10)	C1—C11—H11	120.6
C10—C1—C11	120.73 (10)	C11—N1—N2	119.23 (10)
O1—C2—C1	123.73 (10)	C12—N2—N1	115.18 (10)
O1—C2—C3	115.34 (10)	C12—N2—H2	122.4
C1—C2—C3	120.93 (11)	N1—N2—H2	122.4
C4—C3—C2	120.34 (11)	O2—C12—N2	122.52 (11)
C4—C3—H3	119.8	O2—C12—C13	120.27 (11)
C2—C3—H3	119.8	N2—C12—C13	117.21 (10)
C3—C4—C5	121.30 (11)	C14—C13—C17	118.91 (10)
C3—C4—H4	119.4	C14—C13—C12	125.35 (10)
C5—C4—H4	119.4	C17—C13—C12	115.73 (10)
C6—C5—C4	120.75 (10)	C15—C14—C13	119.05 (11)
C6—C5—C10	120.05 (11)	C15—C14—H14	120.5
C4—C5—C10	119.20 (11)	C13—C14—H14	120.5
C7—C6—C5	121.08 (11)	N3—C15—C14	120.31 (11)
C7—C6—H6	119.5	N3—C15—H15	119.8
C5—C6—H6	119.5	C14—C15—H15	119.8
C6—C7—C8	119.07 (11)	C16—N3—C15	122.24 (10)
C6—C7—H7	120.5	C16—N3—H3A	118.9
C8—C7—H7	120.5	C15—N3—H3A	118.9
C9—C8—C7	121.41 (11)	N3—C16—C17	119.54 (11)
C9—C8—H8	119.3	N3—C16—H16	120.2
C7—C8—H8	119.3	C17—C16—H16	120.2
C8—C9—C10	121.03 (11)	C16—C17—C13	119.85 (11)
C8—C9—H9	119.5	C16—C17—H17	120.1
C10—C9—H9	119.5	C13—C17—H17	120.1
C9—C10—C5	117.31 (10)	O2A—N1A—O3A	121.47 (11)
C9—C10—C1	123.41 (10)	O2A—N1A—O1A	119.66 (10)
C5—C10—C1	119.28 (10)	O3A—N1A—O1A	118.85 (10)
			
C10—C1—C2—O1	178.17 (10)	C11—C1—C10—C9	−1.70 (17)
C11—C1—C2—O1	−0.78 (18)	C2—C1—C10—C5	−0.43 (16)
C10—C1—C2—C3	−1.51 (17)	C11—C1—C10—C5	178.51 (11)
C11—C1—C2—C3	179.55 (11)	C2—C1—C11—N1	3.82 (17)
O1—C2—C3—C4	−178.13 (11)	C10—C1—C11—N1	−175.10 (11)
C1—C2—C3—C4	1.57 (18)	C1—C11—N1—N2	179.91 (10)
C2—C3—C4—C5	0.38 (18)	C11—N1—N2—C12	−179.21 (10)
C3—C4—C5—C6	178.04 (12)	N1—N2—C12—O2	3.65 (17)
C3—C4—C5—C10	−2.30 (18)	N1—N2—C12—C13	−176.03 (9)
C4—C5—C6—C7	178.34 (12)	O2—C12—C13—C14	174.93 (12)
C10—C5—C6—C7	−1.32 (18)	N2—C12—C13—C14	−5.38 (17)
C5—C6—C7—C8	−0.71 (19)	O2—C12—C13—C17	−4.57 (17)
C6—C7—C8—C9	1.9 (2)	N2—C12—C13—C17	175.11 (10)
C7—C8—C9—C10	−1.0 (2)	C17—C13—C14—C15	2.30 (17)
C8—C9—C10—C5	−1.02 (18)	C12—C13—C14—C15	−177.18 (11)
C8—C9—C10—C1	179.19 (12)	C13—C14—C15—N3	−0.78 (18)
C6—C5—C10—C9	2.15 (17)	C14—C15—N3—C16	−2.04 (18)
C4—C5—C10—C9	−177.51 (11)	C15—N3—C16—C17	3.20 (18)
C6—C5—C10—C1	−178.04 (11)	N3—C16—C17—C13	−1.54 (18)
C4—C5—C10—C1	2.30 (17)	C14—C13—C17—C16	−1.17 (18)
C2—C1—C10—C9	179.36 (11)	C12—C13—C17—C16	178.37 (11)

### Hydrogen-bond geometry (Å, °)

**Table:**

*D*—H···*A*	*D*—H	H···*A*	*D*···*A*	*D*—H···*A*
O1—H1···N1	0.84	1.82	2.5519 (15)	145.
N2—H2···O3A	0.88	2.21	3.0332 (19)	155.
N3—H3A···O1A^i^	0.88	1.80	2.6794 (14)	174.
C14—H14···O3A	0.95	2.26	3.1528 (16)	156.
C8—H8···O2^ii^	0.95	2.60	3.2449 (19)	125.
C15—H15···O2A^iii^	0.95	2.61	3.3089 (19)	130.
C16—H16···O1A^iv^	0.95	2.28	3.1923 (16)	160.
C16—H16···O2A^iv^	0.95	2.62	3.4553 (19)	147.

Symmetry codes: (i) −*x*+1, −*y*+2, −*z*+1; (ii) *x*−1, *y*−1, *z*; (iii) *x*, *y*+1, *z*; (iv) *x*+1, *y*+1, *z*.
